# Tunable interlayer excitons and switchable interlayer trions via dynamic near-field cavity

**DOI:** 10.1038/s41377-023-01087-5

**Published:** 2023-03-03

**Authors:** Yeonjeong Koo, Hyeongwoo Lee, Tatiana Ivanova, Ali Kefayati, Vasili Perebeinos, Ekaterina Khestanova, Vasily Kravtsov, Kyoung-Duck Park

**Affiliations:** 1 Department of Physics, Pohang University of Science and Technology (POSTECH), Pohang, 37673 Republic of Korea; 2 School of Physics and Engineering, ITMO University, Saint Petersburg, 197101 Russia; 3 Department of Electrical Engineering, University at Buffalo, New York, NY 14260 USA

**Keywords:** Nanocavities, Nanophotonics and plasmonics

## Abstract

Emerging photo-induced excitonic processes in transition metal dichalcogenide (TMD) heterobilayers, e.g., interplay of intra- and inter-layer excitons and conversion of excitons to trions, allow new opportunities for ultrathin hybrid photonic devices. However, with the associated large degree of spatial heterogeneity, understanding and controlling their complex competing interactions in TMD heterobilayers at the nanoscale remains a challenge. Here, we present an all-round dynamic control of interlayer-excitons and -trions in a WSe_2_/Mo_0.5_ W_0.5_ Se_2_ heterobilayer using multifunctional tip-enhanced photoluminescence (TEPL) spectroscopy with <20 nm spatial resolution. Specifically, we demonstrate the bandgap tunable interlayer excitons and the dynamic interconversion between interlayer-trions and -excitons, through the combinational tip-induced engineering of GPa-scale pressure and plasmonic hot electron injection, with simultaneous spectroscopic TEPL measurements. This unique nano-opto-electro-mechanical control approach provides new strategies for developing versatile nano-excitonic/trionic devices using TMD heterobilayers.

## Abstract (web summary)

We demonstrate an all-round dynamic control of intra- and inter-layer excitonic processes and interconversion between IX and IX− in a WSe_2_/Mo_0.5_ W_0.5_ Se_2_ heterobilayer with <20 nm spatial resolution, achieved by multifunctional tip-enhanced photoluminescence (TEPL) spectroscopy and imaging.

## Graphical abstract

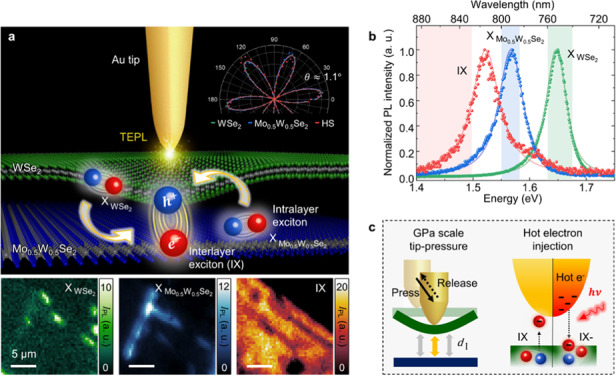

## Introduction

Stacking atomically thin layers of van der Waals (vdW) materials into bilayer heterostructures provides innovative strategies for the development of next-generation optoelectronic devices and substantially broadens the scope of material physics^1–4^. A plethora of intriguing phenomena has been already unveiled in vdW bilayers, but they are likely just the tip of the iceberg because many more structures remain unexplored with different chemical composition, stacking sequence and angle, interlayer distance, and other parameters. Hence, considerable efforts are currently focused on uncovering and controlling the inherent physical properties in vdW heterostructures.

In particular, interlayer excitons (IXs), formed by electrons and holes spatially separated in the top and bottom layers of transition metal dichalcogenide (TMD) heterobilayers^5^, show a range of distinct properties, which are promising for various optoelectronic applications. The reduced spatial overlap of the electron and hole wavefunctions in IXs brings about reduced radiative decay rates, with corresponding lifetimes up to *μ*s^6^, while the interlayer distance and twist angle between the constituent monolayers provide knobs for tuning the IX quantum yield^7^. In addition, the out-of-plane component of the IX dipole moment enables straightforward electric field control. IXs in TMD heterobilayers also provide long-lived valley polarization and coherence^8^, circumventing the limits of TMD monolayers and enabling practical valleytronic applications. Additionally, the slight lattice mismatch and twist angles in heterobilayers give rise to moiré supperlattices and corresponding confinement potentials that can effectively trap IXs^9^. Therefore, IXs in TMD heterobilayers provide a promising element for realizing excitonic integrated circuits^10,11^ and possibly demonstrating high temperature many-body effects, such as Bose-Einstein condensates (BEC) and superfluidity^12^. In addition, since trions provide further opportunities for electrostatic control in excitonic circuits as well as longer radiative lifetimes compared to neutral excitons^13^, inducing and controlling the interlayer trions (IX − or IX + ) in TMD heterobilayers are highly desirable.

However, in order to enable practical applications of TMD heterostructures using IX and IX − , several major challenges must be overcome, one of which is the large degree of spatial heterogeneity. The underlying processes, e.g., competing interactions of coupling, dephasing, and energy transfer of intra- and inter-layer excitons as well as IX− interconversion, arise at the nanoscale and cannot be understood by diffraction-limited optical approaches, calling for the near-field optical probing^14–17^. Furthermore, beyond probing, it is highly important to achieve nanoscale control of local IX and IX− properties in TMD heterostructures. However, dynamic control study of nanoscale properties of IXs with simultaneous nano-spectroscopic measurements has rarely been reported^18,19^ and the more desirable interlayer trion-conversion has not been investigated yet.

Here, we demonstrate an all-round dynamic control of intra- and inter-layer excitonic processes and interconversion between IX and IX− in a WSe_2_/Mo_0.5_ W_0.5_ Se_2_ heterobilayer with <20 nm spatial resolution, achieved by multifunctional tip-enhanced photoluminescence (TEPL) spectroscopy and imaging. The use of the alloy-based heterobilayer allows us to tune the bandgap energy of IX and X depending on the molecular compositions. We optimize the W/Mo composition of 0.5 to induce strong electronic resonance between the tip-plasmon and the intra- and inter-layer exciton PLs while remaining well separated^20^. Through hyperspectral TEPL nano-imaging, we reveal nanoscale inhomogeneities of the IX emission and identify the regions with the different interlayer coupling strength. At the weak interlayer coupling region, we dynamically control the radiative recombination path and competing emission rates of intra- and inter-layer excitons through the engineering of Au tip-heterobilayer distance and interlayer distance, achieving increase of the IX quantum yield compared to that of intralayer excitons (Xs).

In addition, by applying GPa scale tip-pressure to the heterobilayer, we directly modify its electronic bandstructure, which is demonstrated via blueshifted IX TEPL energy and supported by theoretical calculations. Furthermore, through the control of plasmonic hot electron injection from the Au tip, we convert neutral IXs into charged IX− states. This approach presents the first-ever scanning-tip hot electron regulator, which can precisely control the hot electron transfer rate or trion-conversion rate in a fully reversible fashion. Our results demonstrate that IX and IX − in TMD heterobilayers can be accurately controlled in nanoscopic volumes via a near-field approach, which opens up new avenues for the development of compact TMD based optoelectronic devices and provides insights for studying various many-body phenomena.

## Results and discussion

### Experimental configuration for TEPL spectroscopy

**Fig. 1:**
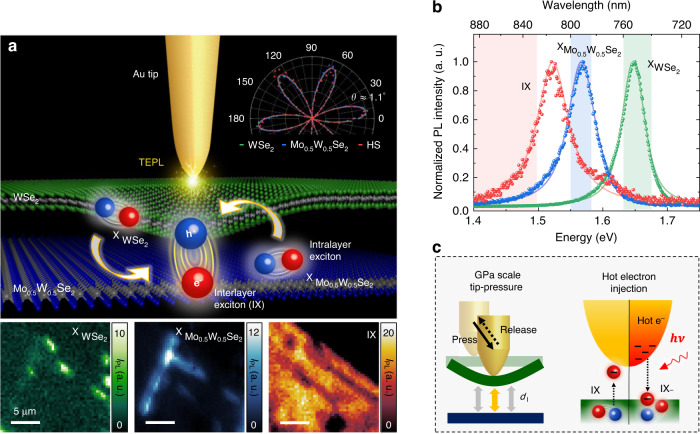
Schematic of multifunctional tip-enhanced photoluminescence spectroscopy to dynamically control excitonic processes in TMD heterobilayer. **a** (Top) Illustration of the WSe_2_/Mo_0.5_W_0.5_Se_2_ heterobilayer with SHG polarization dependence and the Au tip to probe and control the crystal. (Bottom) Hyperspectral confocal PL images of the heterobilayer for the integrated intensities 740–760 nm (Green, ${\rm{X}}_{{\rm{WSe}}_{2}}$), 784–800 nm (Blue, ${\rm{X}}_{{\rm{Mo}}_{0.5}{\rm{W}}_{0.5}{\rm{Se}}_{2}}$), and 830–900 nm (Red, IX). Scale bars are 5 μm. **b** Normalized far-field PL spectra of IX, ${\rm{X}}_{{\rm{Mo}}_{0.5}{\rm{W}}_{0.5}{\rm{Se}}_{2}}$, and ${\rm{X}}_{{\rm{WSe}}_{2}}$. Red, blue, and green colored regions are spectral regions used for hyperspectral images in (**a**), respectively. **c** Schematic diagram describing the multifunction of TEPL spectroscopy to dynamically control the intralayer- and interlayer-excitonic properties, such as interlayer coupling strength and interlayer trion (IX − ) conversion

We fabricate a WSe_2_/Mo_0.5_ W_0.5_ Se_2_ heterobilayer on a Au film by stacking exfoliated ML flakes with their crystal axes aligned for optimized IX emission. The twist angle is measured to be ~1.1^o^ via polarization-resolved second-harmonic generation (SHG) spectroscopy (see inset of Fig. 1a and Methods). In the assembled heterobilayer, IXs are formed by the spatially separated holes (h^+^) and electrons (e^−^) in constituent layers in addition to intralayer excitons ${\rm{X}}_{{\rm{WSe}}_{2}}$ and ${\rm{X}}_{{\rm{Mo}}_{0.5}{\rm{W}}_{0.5}{\rm{Se}}_{2}}$. Hyperspectral far-field PL imaging shows that the spatial distributions of intra- and inter-layer excitons are considerably inhomogeneous at the microscale, as shown in Fig. 1a (see Fig. S1 for the optical microscopy image). Such spatial heterogeneity in vdW heterostructures is attributed to the non-uniform interlayer coupling strength, which depends sensitively on local strain-induced deformation and interfacial contamination^21,22^. Furthermore, on the smaller spatial scales below the diffraction-limit, nanoscale structural deformations, such as wrinkles, bubbles, and grain boundaries^23–25^, give rise to complex charge dynamics and interactions with competing recombination processes of intra- and inter-layer excitons.

To develop comprehensive understanding of the nanoscale heterogeneity in the WSe_2_/Mo_0.5_ W_0.5_ Se_2_ heterobilayer and demonstrate its precise control, we develop multifunctional TEPL spectroscopy. We use a radially polarized excitation beam in the bottom-illumination geometry to induce strong out-of-plane optical fields and plasmons at the Au tip-Au film junction (see Methods for more details). The tip plasmons induced by the dipole-dipole interaction between the Au tip and the Au substrate then couple with the Xs and IXs (Fig. 1b) in the heterobilayer and enhance their PL responses via the Purcell effect^26^. The tip-sample distance is regulated with a precision of ~0.2 nm using a shear-force feedback loop, with corresponding control on the plasmon enhancement and optical field strength. This allows us to dynamically manipulate the light-matter interactions at the nanoscale with the simultaneous spectroscopic TEPL measurements. Fig. 1c shows a schematic of the TEPL spectroscopy, including different multifunctional control modalities, i.e., GPa scale tip-pressure and plasmonic hot carrier injection, as well as tip-induced engineering of the interlayer distance (*d*_I_) in a TMD heterobilayer.

### Near-field probing of the nanoscale heterogeneity in a TMD heterobilayer

**Fig. 2:**
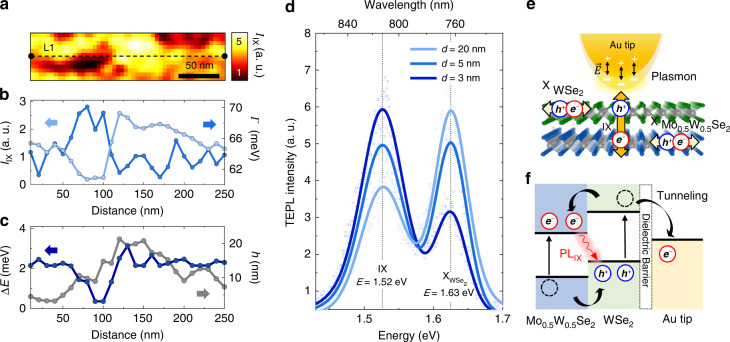
Nanoscale heterogeneity of the interlayer coupling strength and TEPL of IX and ${\rm{X}}_{{\rm{WSe}}_{2}}$. **a** Hyperspectral TEPL image of the heterobilayer exhibiting inhomogeneous IX emission with 20 nm spatial resolution. **b**–**c** Spectroscopic and topographic line profiles for the dashed line L1 in (a). Nanoscale spatial heterogeneities in TEPL peak intensity *I*_IX_, linewidth *Γ*, peak energy shift Δ*E*, and topographic height ℎ are revealed far beyond the diffraction limit. **d** Evolving TEPL spectra of the heterobilayer as a function of the tip-sample distance *d*. The PL responses of IX (*E* = 1.52 eV) and WSe_2_ (*E* = 1.63 eV) are acquired with the tip located in the weak interlayer coupling region. **e** Illustration for the more efficient plasmon-IX (out-of-plane dipole) coupling compared to the plasmon-X (in-plane dipole) coupling when the Au tip closely approaches to the crystal. **f** Illustration for the type-II band alignment of a WSe_2_/Mo_0.5_ W_0.5_ Se_2_ heterobilayer and the work function of Au tip describing the charge transport mechanisms. This energy transfer mechanism explains our experimental results of increased (decreased) TEPL intensity of interlayer (intralayer) excitons when the tip approaches to the heterobilayer

To investigate the nanoscale heterogeneity of IXs originated from the non-uniform interlayer coupling strength, we perform hyperspectral TEPL imaging of the heterobilayer, with the experimentally observed spatial distribution of the tip-enhanced IX PL as shown in Fig. 2a. In our TEPL scanning, the tip-sample distance *d* is kept at ~5 nm to minimize tip-induced modification of the sample surface. To better visualize the spectroscopic information of the inhomogeneous IX distribution and corresponding topography, in Fig. 2b and c we present the TEPL intensity *I*_IX_, peak energy shift Δ*E* (Δ*E* = 0 at *E* = 1.52 eV), linewidth *Γ*, and height ℎ along the line L1 (indicated in Fig. 2a). The variations in *I*_IX_ indicate the non-uniform interlayer coupling strength and associated possible changes in the density, oscillator strength, and emission lifetimes of IXs.

The regions with higher *I*_IX_ generally show narrower *Γ* and blueshifted peak energy. The broader *Γ* in the low-density IX regions is possibly due to the slight deviation of the IX dipole orientation, since it can cause PL energy variation due to the quantum confinement effect on the interlayer excitonic properties^27^. The observed blueshift of the high-density IXs is originated from the static electric dipole of IX because the repulsive interactions between the well-oriented IXs cause a mean-field shift, as revealed in previous far-field studies^11^. Note that the height ℎ generally shows an uncorrelated behavior with the spectroscopic line profiles which means the interlayer coupling strength is not simply characterized by the surface profiling^21,28^. It should be noted that the whole region of Fig. 2a is measured with ~20 nm spatial resolution by TEPL imaging, which is much smaller than the diffraction-limited beam spot size. Hence, the observed spatio-spectral heterogeneity cannot be investigated using a conventional far-field imaging methods, such as confocal microscopy (see Fig. S2 for the confocal PL image of the same measured area).

We then position the tip in the weak interlayer coupling region and acquire different PL characteristics of IX and ${\rm{X}}_{{\rm{WSe}}_{2}}$ as a function of the tip-sample distance *d* for selected distances, as shown in Fig. 2d. At *d* = 20 nm, we observe far-field PL spectrum exhibiting IX and ${\rm{X}}_{{\rm{WSe}}_{2}}$ peaks at *E* = 1.52 eV and 1.63 eV, respectively. The PL peak of ${\rm{X}}_{{\rm{Mo}}_{0.5}{\rm{W}}_{0.5}{\rm{Se}}_{2}}$ is not clearly observed due to its low quantum yield. At this relatively large tip-sample distance, the ${\rm{X}}_{{\rm{WSe}}_{2}}$ peak shows higher PL intensity than the IX peak due to the low interlayer coupling strength. In comparison, at *d* = 5 nm the intensities of the ${\rm{X}}_{{\rm{WSe}}_{2}}$ and IX PL become similar. Here, the plasmon-exciton coupling in the Au tip-Au film nanocavity is significantly stronger, and since the plasmonic resonance predominantly enhances out-of-plane optical fields, the PL of the vertically oriented IX dipoles is increased^26^. At the same time, the intralayer excitons, while efficiently excited in the far-field via in-plane polarized fields, show increasingly inhibited PL emission inside the plasmonic cavity at smaller distances *d*. When the tip approaches closely to the heterobilayer with *d* = 3 nm, the PL intensities of the ${\rm{X}}_{{\rm{WSe}}_{2}}$ and IX peaks are switched, and the IX emission dominates, while its spectral position and shape remain unchanged (see Fig. S3 for optical field distributions at the Au tip).

The TEPL enhancement of IXs is attributed to the increased excitation rate and Purcell effect (see the Supplementary information Section 4 for the calculated enhancement factor ~1.6 × 10^3^)^26^. In addition, the tip-induced charge tunneling effect further influences the observed TEPL responses of IXs and Xs^29,30^. In the near-field regime approaching tip-sample contact, the effective overlap between electron wavefunctions of the Au tip and the heterobilayer can facilitate charge tunneling processes^31^ that cause the perturbation of the excitonic system. Fig. 2f illustrates the charge transport mechanism of the type-II band alignment when the tip approaches the 2D crystal surface. Since the Fermi level of Au lies lower than the conduction band minimum energy in WSe_2_, the electrons at the adjacent WSe_2_ tunnel into the Au tip. Additionally, the electrons and holes in the heterobilayer are redistributed via interlayer charge transfer. Consequently, the *p*-doped top layer and the *n*-doped bottom layer effectively facilitate the IX recombination at the local region with decreasing recombination rate of intralayer excitons, as experimentally confirmed in the result of Fig. 2d.

### Tip-induced nano-engineering of TMD heterobilayer

**Fig. 3:**
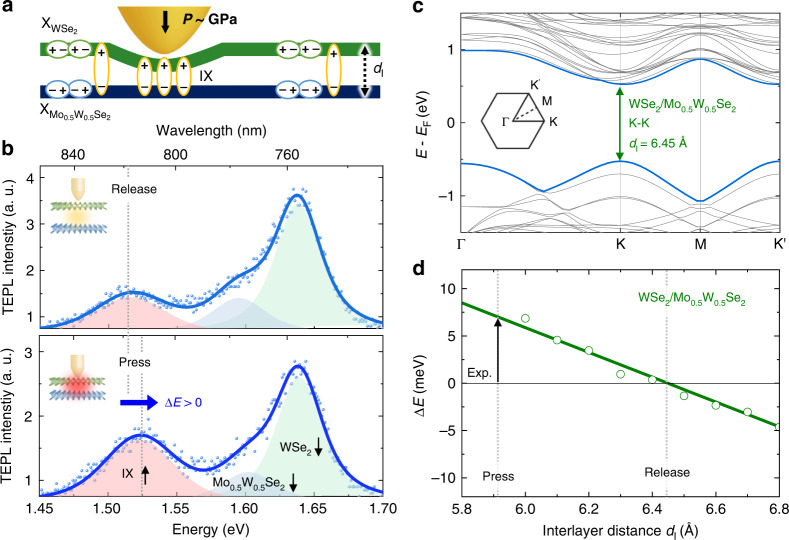
Tip-induced control of interlayer coupling strength and simulated electronic bandstructures. **a** Schematic illustration of the local tip control of the interlayer distance *d*_I_ in a WSe_2_/Mo_0.5_W_0.5_Se_2_ heterobilayer. **b** TEPL spectra before (top) and after (bottom) pressing the heterobilayer with GPa scale tip-pressure, which causes significant modifications in electronic bandstructure. **c** Electronic bandstructure calculated via DFT for a WSe_2_/Mo_0.5_ W_0.5_ Se_2_ heterobilayer (gray) at equilibrium interlayer distance *d*_I_ = 6.45 Å, with the highest valence and lowest conduction bands highlighted in blue; the direct K-K transition is indicated with green arrow; inset shows high-symmetry points in the hexagonal Brillouin zone. The Fermi energy *E*_F_ is defined as an averaged energy of the conduction band minimum and the valence band maximum. **d** Calculated energy shift for the K-K transition in a WSe_2_/Mo_0.5_ W_0.5_ Se_2_ heterobilayer as a function of *d*_*I*_ (green circles) and corresponding linear fit (green line), together with the experimentally measured tip-induced IX shift (black arrow)

In order to move towards practical opto-electronic device applications of vdW heterobilayers, the nanoscale heterogeneity of IX and X emission should be not only resolved, but also actively controlled. Recently, a few approaches for engineering local exciton properties in 2D heterostructures were demonstrated, for example, via electrostatic field^10,32^ or high magnetic field^33^. However, precise nanoscale control of emission beyond the tip-sample distance modulation is a significant challenge^18,34^. To further extend our tip-induced IX emission control, we present a nano-opto-mechanical tip-pressure engineering approach through the atomic force tip control combined with in-situ TEPL spectroscopy. As schematically illustrated in Fig. 3a, the tip exerts local pressure within a ~25 nm^2^ sample area, which is precisely regulated through changing the set-point in a shear-force feedback loop (see Methods). This pressure is expected to cause a local decrease in the interlayer distance and corresponding increase in the interlayer coupling strength. We experimentally verify this behavior by measuring TEPL spectra evolution in a reversible tip-press and -release process. As we demonstrate in Fig. S5 in the Supplementary information, tip pressure applied to a sample region with initially weak interlayer coupling results in stronger IX emission with simultaneously decreased X emission of WSe_2_ and Mo_0.5_ W_0.5_ Se_2_ which is attributed to the improved interlayer coupling strength^35,36^.

In our previous study, we demonstrated that tip-induced local pressure can exceed 10 GPa owing to its nanoscale tip-sample contact area even though the tip-force is only on the order of pN^21,37^. Here, in the same fashion we induce ~GPa scale tip-pressure in a TMD heterobilayer (see the Supplementary information Section 6 and 7 for the estimation of pressure and compressive strain), which directly modifies its crystal structure and electronic bandstructure, resulting in the modified IX emission properties. Fig. 3b shows the modified TEPL spectra before (top panel) and after (bottom panel) inducing ~GPa scale tip-pressure in the WSe_2_/Mo_0.5_ W_0.5_ Se_2_ heterobilayer. The spectra are decomposed into 3 peaks corresponding to IX, ${\rm{X}}_{{\rm{WSe}}_{2}}$, and ${\rm{X}}_{{\rm{Mo}}_{0.5}{\rm{W}}_{0.5}{\rm{Se}}_{2}}$ via fitting by Lorentzian functions. In addition to the increase in the IX/X PL ratio discussed earlier, the IX TEPL peak exhibits a clearly discernible blueshift of ~7 meV. In order to clarify the physical origin of the observed spectral changes, we simulate the associated electronic bandstructure modification with decreasing interlayer distance using density functional theory (DFT) calculations with 2 × 2 supercell as described in Methods.

The calculated electronic bandstructure for the experimentally studied WSe_2_/Mo_0.5_W_0.5_Se_2_ heterobilayer in the equilibrium configuration with AA’ stacking is shown in Fig. 3c. This configuration corresponds to the interlayer distance *d*_I_ = 6.45 Å, which is defined as the separation between the transition atom planes in the WSe_2_ and Mo_0.5_W_0.5_Se_2_ layers and determined numerically by relaxing the structure in DFT calculations using empirical van der Waals interactions^38^. The highest valence band and the lowest conduction band are highlighted with blue color and exhibit their maximum and minimum energy, respectively, at the K high-symmetry point (see schematic of the hexagonal Brillouin zone corresponding to the 2 × 2 supercell in the inset).

To study the effect of tip-induced strain, we calculate bandstructures for different values of interlayer distance *d*_I_ and extract the corresponding energies of the K-K electronic transition. The results are plotted in Fig. 3d as shifts of the K-K transition energy from that at the equilibrium interlayer distance *d*_I_ = 6.45 Å (green circles). In the experimentally relevant range of energy shifts >10 meV, the dependence on interlayer distance is well described by a linear fit (green line). Comparison with the experimentally observed IX energy blueshift of ~7 meV (marked with black arrow in Fig. 3d) provides an estimate for the interlayer distance decrease in the maximally strained configuration of Δ*d*_I_~0.6 Å relative to the equilibrium interlayer distance. Assuming a typical force constant per unit area for the vibrational breathing modes^39^ of a TMD heterobilayer of *K*_z_ = 8.6 × 10^19^ N/m^3^, we further estimate the tip-induced pressure as *P* = *K*_z_ · Δ*d*_I_ ≈ 5 GPa, which is of the same order of magnitude as values estimated from modelling in the Supplementary information Section 7 and reported previously^37^.

We note that the IX energy shift induced by compressive strain is sensitive to the chemical composition of the TMD bilayer. Our calculations for pristine bilayers (see the Supplementary information Section 8) show that similar tip-induced pressure would result in a higher ~20 meV blueshift in a WSe_2_/MoSe_2_ heterobilayer, while for WSe_2_/WSe_2_ homobilayer it would result in ~20 meV redshift. Therefore, our estimates of the applied pressure can be in general affected by fluctuations in local stoichiometry. Furthermore, our calculations do not account for local inhomogeneities of the initial strain or strain-dependent binding energies of IX states^40^, thus providing only an order-of-magnitude estimate of the induced strain.

### Tip-induced hot electron injection control of charged IX

**Fig. 4:**
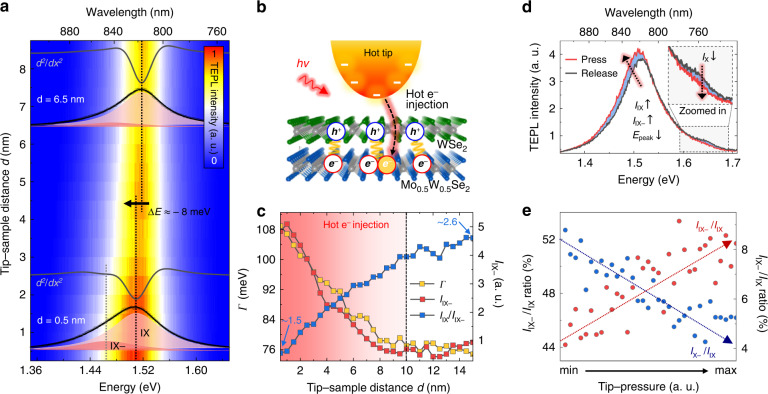
Inducing and controlling of IX− via hot electron injection of the Au tip. **a** TEPL spectra of the heterobilayer with respect to the tip-sample distance *d* at the high excitation intensity (≈10^9^ W/m^2^) exhibiting apparent emergence of the IX− shoulder. For the selected TEPL spectra at *d* = 6.5 nm and *d* = 0.5 nm, interlayer exciton (IX, black dashed line) and interlayer trion (IX−, light gray dashed line) peaks are assigned by the corresponding dips in the second derivative of the TEPL spectra (*d*^2^/*d*_*x*_^2^, gray curves) fitted with a Lorentzian function. **b** Illustration for the tip-induced hot electron injection process, which stimulates the IX− generation in the heterobilayer. **c** Changes of the linewidth (*Γ*, yellow), IX intensity (*I*_IX−_, red), and intensity ratio of IX/IX− (*I*_IX_/*I*_IX−_, blue) as a function of *d*. *Γ*, *I*_IX−_, and *I*_IX_/*I*_IX−_ are directly derived from the TEPL spectra. **d** TEPL spectra when the Au tip presses (red) and releases (black) the heterobilayer exhibiting further enhancement of IX− emission via GPa scale tip-pressure. **e** Changes of TEPL intensity ratio for *I*_IX−_/*I*_IX_ (red) and *I*_X_/*I*_IX_ (blue) when the Au tip presses the crystal

The experimental results reported so far have been measured at relatively low values of excitation intensity (≈10^8^ W/m^2^). By significantly increasing the excitation intensity, we can explore a different regime, characterized by electron transport from the Au tip to the heterobilayer, which is due to the hot electron generation at the plasmonic tip and subsequent injection into the Mo_0.5_ W_0.5_ Se_2_ ML^41,42^. Our measurements of the excitation-intensity dependent IX PL confirm the increased charged interlayer exciton (IX−) density in contrast to the saturating neutral IX density at the high-intensity regime attributed to the hot carrier injection^43,44^ (see the Supplementary information Section 9 for more details). By approaching the plasmonic ℎ*ot tip* with a strongly localized field close to the heterobilayer, we achieve the dynamic local control of the IX− formation and recombination rate in the near-field regime. To demonstrate such control, we move to the sample region where the dominant IX emission is observed without the X emission and investigate the tip-induced hot e^−^ injection effect at the high excitation intensity (≈ 10^9^ W/m^2^). Fig. 4a shows TEPL spectra of IX peak with respect to the tip-sample distance *d*, exhibiting apparent emergence of the IX− peak when the Au tip closes to the crystal. To quantify the IX and IX− peak energies for the TEPL spectrum obtained at *d* = 0.5 nm, we deconvolute the spectrum by the Lorentzian function. The fitted spectrum and its second derivative curve (*d*^2^ /*d*_*x*_^2^, gray) clearly show the pronounced IX− peak at ~1.465 eV, in addition to the neutral IX peak at *E* = ~1.509 eV, which is attributed to the hot e^−^ injection. By contrast, the IX− peak is rarely seen in the TEPL spectrum at *d* = 6.5 nm due to the low hot e^−^ injection efficiency. Through the deconvolution of TEPL spectrum, we can derive the IX and IX− peak energies of ~1.518 eV and ~1.474 eV. However, the energy assignment for the IX− is not clearly seen in the second derivative curve due to the nonsignificant IX− shoulder. Note that, from the comparison of IX peak energies at *d* = 6.5 nm and *d* = 0.5 nm, we find the TEPL redshift of ~8 meV. This redshift is also supporting evidence of the hot e^−^ injection because photo-induced doping in TMD crystals gives rise to PL energy redshift.

The mechanism of the tip-induced IX− generation is schematically illustrated in Fig. 4b. The hot electrons injected into the heterobilayer within the nanoscale region under the plasmonic tip couple with the neutral IX and form the IX−. Since this process becomes increasingly efficient as the tip approaches the heterobilayer, the local density of the neutral IX shows saturating feature (see the Supplementary information Section 9 for more details). To demonstrate this behavior from the result of Fig. 4a, we plot the changes of the linewidth (*Γ*, yellow), IX− intensity (*I*_IX−_, red), and intensity ratio of IX/IX− (*I*_IX_/*I*_IX−_, blue) as a function of *d*, as shown in Fig. 4c. Note that, *Γ*, *I*_IX−_, and *I*_IX_/*I*_IX−_ are directly derived from the experimentally observed TEPL spectra. As seen in the figure, *Γ* and *I*_IX−_ start to increase at *d* ≈ 10 nm, which means the threshold distance for the hot e^−^ -induced IX− generation. This distance is in good agreement with the results of previous surface-enhanced Raman spectroscopy studies for molecular samples^45^. In addition, the abruptly decreasing *I*_IX_/*I*_IX−_ from *d* = 10 nm, also indicates a highly efficient conversion from the neutral IX to IX− under the plasmonic tip as expected.

We further enhance and control the charged IX emission by applying GPa scale pressure with the tip under the high-power excitation. As shown in Fig. 4d, under the tip-induced pressure the contributions to the total TEPL intensity from both IX− and IX peaks are increased, which we attribute to the higher interlayer coupling strength and correspondingly increased recombination rate for both neutral and charged IX species. This is accompanied by redshift of the IX TEPL spectrum and increased linewidth, which contrasts with the observed blueshift of the TEPL spectrum at low excitation powers presented in Fig. 3b. Additionally, we observe that the TEPL intensity of intralayer excitons (*I*_X_ for a WSe_2_ ML) is decreased, which is naturally understood from the competing recombination process between the intra- and inter-layer excitons, as discussed earlier regarding the data presented in Fig. 3. Furthermore, the precise modification of IX, IX− and X emissions is clearly demonstrated in Fig. 4e. When we press the sample with GPa scale tip pressure, the TEPL intensity ratios for IX−/IX (red) and ${\rm{X}}_{{\rm{WSe}}_{2}}$ /IX (blue) show opposite behaviors with the pressure. This result shows a distinct advantage of our work compared to the previous hot e^−^ injection studies, i.e., the ability to dynamically control the hot e^−^ density and the corresponding IX− conversion rate. By regulating the tip-sample distance precisely (~0.2 nm^26^) using the scanning probe tip, we can control the hot e^−^ injection at the nanoscale in a fully reversible manner, which was not possible in the previous studies^46,47^ (see Fig. S10 for demonstration of reversible control).

## Conclusion

In summary, we have investigated the nanoscale heterogeneity of the interlayer coupling strength in an aligned WSe_2_/Mo_0.5_ W_0.5_ Se_2_ heterobilayer and demonstrated active control of its emission via multifunctional TEPL spectroscopy inside a plasmonic tip-substrate cavity in two distinct power regimes. At low excitation powers, we control the interplay between the intralayer and neutral interlayer exciton PL via distance-tunable Purcell enhancement, where IX emission becomes dominant at small tip-sample distances. At high excitation powers, the plasmonic tip acts as a source of hot electrons, which are injected into the heterobilayer and facilitate formation of IX− with distance-tunable efficiency. Beyond the simple control of interlayer excitons via tip-sample distance modulation, we reversibly modify their spectral response via applying nano-localized tip-induced GPa scale pressure. We support the observed local pressure-dependent IX spectral evolution with DFT simulations, which provide insights into interlayer distance dependent band structure in aligned TMD bilayers. The presented results demonstrate new approaches to study the nanoscale heterogeneity of the IX response in TMD heterobilayers and suggest ways to control that response within nanoscopic sample areas. This manifests an important step towards the development of next-generation optoelectronic devices, such as nano-integrated excitonic/trionic circuits, and investigation of novel many-body effects with TMD-based heterobilayers.

## Methods

### Sample preparation

Cover glass (170 *μ*m thickness) was ultrasonicated in acetone and isopropanol for 10 min each and cleaned again by O_2_ plasma treatment for 10 min. Then, a Cr adhesion layer (2 nm thickness) and an Au film (9 nm thickness) were deposited subsequently on the glass with a rate of 0.1 Å/s each at the base pressure of ~10^−6^ torr using a conventional thermal evaporator. The prepared substrate was covered with a 0.5 nm thick layer of Al_2_O_3_ via atomic layer deposition to reduce the PL quenching with maintaining the large TEPL enhancement. TMD monolayers (Mo_0.5_ W_0.5_ Se_2_, WSe_2_) were mechanically exfoliated from corresponding bulk crystals (HQ Graphene) onto polydimethylsiloxane (PDMS) stamps. For better homogeneity of the target heterobilayer, the monolayers were exposed to UV light^48^ for 10 min. To achieve accurate layer alignment in the heterobilayer, the directions of crystallographic axes for the monolayers were determined from polarization-resolved second-harmonic generation (SHG) measurements under excitation with laser pulses of 1200 nm center wavelength and 100 fs duration. The WSe_2_ and Mo_0.5_ W_0.5_ Se_2_ monolayers were then stacked together on a PDMS substrate with their crystallographic axes aligned via dry transfer at a temperature of 60 °C. The twist angle between the monolayers in the resulting heterobilayer was measured again with polarization-resolved SHG. Finally, the heterobilayer was placed onto the Au-covered substrate for near-field measurements via dry transfer.

### Multifunctional TEPL spectroscopy and imaging setup

Multifunctional TEPL spectroscopy is based on the bottom-illumination mode confocal optics setup combined with shear-force AFM using the Au tip. For the excitation beam, He-Ne laser (*λ* = 632.8 nm, optical power *P* of ≤0.5 mW) was passed through a radial polarizer and then focused at the Au tip-Au film junction by an oil immersion objective lens (PLN100x, 1.25 NA, Olympus). The radial polarizer was used to make vertically polarized beam component as large as possible at the tip apex which leads to effective coupling of exciton and cavity plasmon inducing highly enhanced TEPL signals. The backscattered TEPL signals from a sample were collected by the same objective lens. Note that we use high NA objective lens for efficient collection of the interlayer exciton emissions which has out-of-plane dipole moment. In addition, undesirable far-field background noise was reduced by using a pinhole in the detection scheme. TEPL signals (633 nm cut-off) were then sent to a spectrometer (f = 320 mm, 150 g/mm, ~1.6 nm spectral resolution, Monora320i, Dongwoo Optron) and finally imaged onto a thermoelectrically cooled charge-coupled device (CCD, DU971-BV, Andor) to obtain TEPL spectra. For hyperspectral nano-imaging, TEPL spectra at each pixel were recorded during an AFM scanning by a digital controller (Solver next SPM controller, NT-MDT) based on the Au tip attached on a quartz tuning fork. The Au tip (apex radius of ~10 nm) was prepared by the refined electrochemical etching protocol^49^ and attached to a tuning fork with a super glue. The tip-sample distance was regulated by the shear-force feedback through monitoring the changing dithering amplitude of the tuning fork/tip assembly.

### Tip-induced pressure-engineering of heterobilayer

To perform the nanoscale pressure-engineering of the heterobilayer using the Au tip, we gradually changed the setpoint of the shear-force feedback. To modify the electronic bandstructure (Fig. 3), we gradually lowered the setpoint to ~75% of the initial oscillating amplitude to induce ~GPa pressure to the crystal structures.

### Simulations of electronic bandstructures in TMD hetero- and homo-bilayers

For this study, we performed first-principle calculations for the geometry optimization and electronic band structures using density functional theory (DFT) implemented in Quantum ESPRESSO^50^. The electronic exchange-correlation interactions were treated using generalized gradient approximation (GGA) with the method of Perdew-Burke-Ernzerhof (PBE)^51^. The optimized norm-conserving Vanderbilt fully relativistic pseudopotentials^52^ from the PseudoDojo library [http://www.pseudo-dojo.org/] were used. The Brillouin zone was sampled by a 18 × 18 × 1 Monkhorst-Pack k-point mesh for all calculations in a 2 × 2 supercell. The kinetic energy cut-off was 70 Ry. The full geometry optimizations were performed with energy and force tolerances of 10^−6^ eV and 10^−6^ eV Å^−1^, respectively. To simulate the hydrostatic pressure, we fixed metal atoms in two planes separated by a fixed distance *d*_I_ and relaxed vertical positions of Se atoms. A vacuum space of 20 Å was considered between the bilayers to avoid any interaction between them. To account for the interlayer vdW interactions, we used vdW density functionals^53^ for all the simulations.

## Supplementary information

Supplimentary information

## Data Availability

The data that support the findings of this study are available from the corresponding author upon reasonable request.
