# Supplementary material for: Tunable interlayer excitons and switchable interlayer trions via dynamic near-field cavity
Source: Light Sci Appl. 2023 Mar 3;12:59. doi: 10.1038/s41377-023-01087-5 (PMC9981773; doi:10.1038/s41377-023-01087-5)
Supplement: Supplementary file 1 — Supplimentary information [file 41377_2023_1087_MOESM1_ESM.docx]

Supplementary Information for

Tunable interlayer excitons and switchable interlayer trions via dynamic near-field cavity

Yeonjeong Koo^1#^, Hyeongwoo Lee^1#^, Tatiana Ivanova^2^, Ali Kefayati^3^, Vasili Perebeinos^3^, Ekaterina Khestanova^2^, Vasily Kravtsov^2*^, and Kyoung-Duck Park^1*^

*^1^Department of Physics, Pohang University of Science and Technology (POSTECH), Pohang 37673, Korea*

*^2^School of Physics and Engineering, ITMO University, Saint Petersburg 197101, Russia*

*^3^Department of Electrical Engineering, University at Buffalo, The State University of New York, Buffalo, New York 14260, United States*

*^#^ These authors contributed equally to this work*

** Corresponding authors*

*Email:* [*vasily.kravtsov@metalab.ifmo.ru*](mailto:vasily.kravtsov@metalab.ifmo.ru)*,* [*parklab@postech.ac.kr*](mailto:parklab@postech.ac.kr)

1. Optical image of WSe_2_/Mo_0.5_W_0.5_Se_2_ heterobilayer


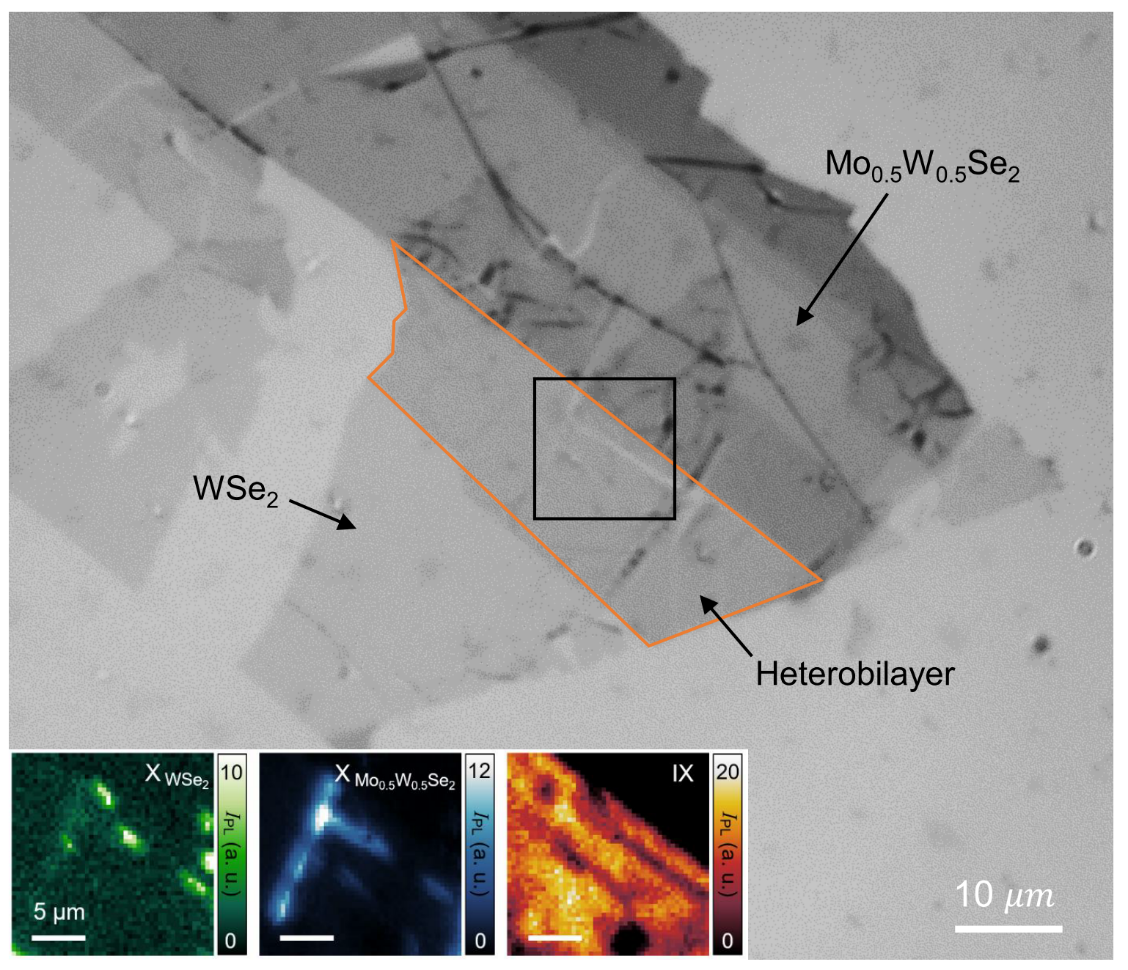


**Fig. S1.** Optical image of a WSe_2_/Mo_0.5_W_0.5_Se_2_ heterobilayer. Far-field hyperspectral PL images of an indicated area (black solid box) are presented in Fig. 1a.

2. Comparison to confocal photoluminescence imaging

For direct comparison, we measured a photoluminescence (PL) image via confocal microscopy for the same measurement region as the hyperspectral TEPL image in Fig. 2a in the main text. As shown in Fig. S2, in the far-field PL image the nanoscale non-uniform interlayer coupling strength and associated possible variations of IXs cannot be resolved. This result shows the superior resolving power of TEPL imaging compared to the conventional confocal PL imaging.


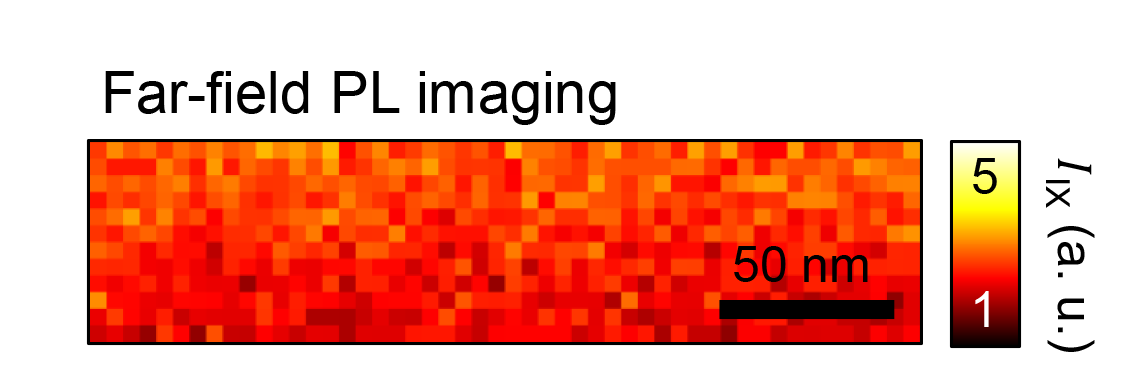


**Fig. S2.** Far-field PL image for the same region as shown in Fig. 2a in the main text. Here, the nanoscale excitonic properties cannot be resolved due to the diffraction limited spatial resolution.

3. In-plane and out-of-plane optical field distributions at plasmonic nanotip

Fig. S3a and b show that the local optical field enhancement of the out-of-plane mode is stronger by a factor of $\geq$3$\times$10^2^ compared to the in-plane mode right below the Au tip apex. Because the in-plane dipole of the X_WSe2_ couples to the gap-plasmon oscillation of the Au tip and Au substrate very inefficiently, nonradiative damping and PL quenching processes dominate over enhancement at few nm gaps. In contrast, with the out-of-plane dipole of the IX efficiently coupled to the tip-substrate gap plasmon, its radiative decay is increasingly enhanced at smaller gaps, which leads to the evidently enhanced IX TEPL signal as shown in Fig. 2d in the main text.


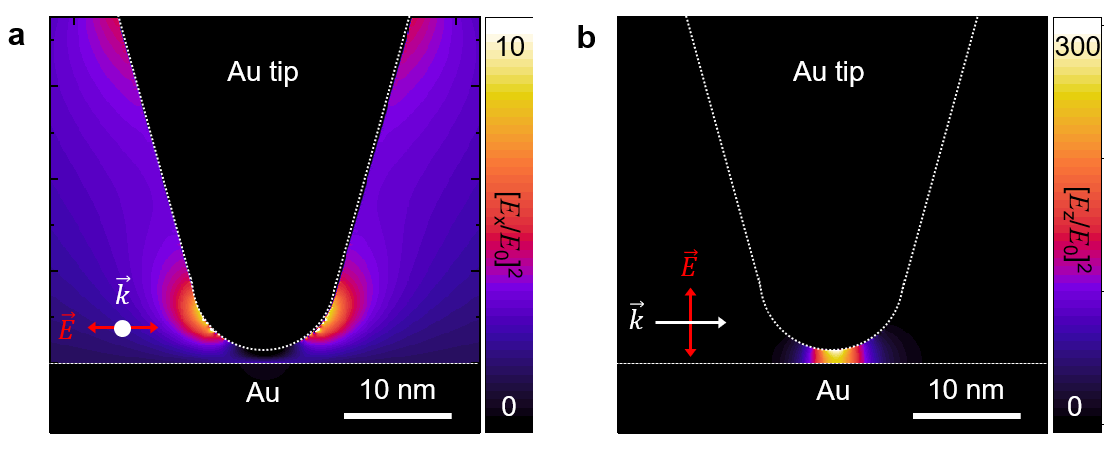


**Fig. S3.** Results of finite-difference time domain (FDTD) simulations for the in-plane (**a**) and out-of-plane (**b**) optical field intensity and confinement under the experimental conditions of tip-induced pressure engineering with simultaneous TEPL spectroscopy.

4. Calculation of TEPL enhancement factor

For the experimental result obtained on a WSe_2_/Mo_0.5_W_0.5_Se_2_ heterobilayer as shown in Fig. 2a of the main text, we can use the following equation to estimate the TEPL enhancement factor (*EF*) [1]:

$$EF=\left( \frac{I_{tip-in}-I_{tip-out}}{I_{tip-out}} \right)\times\frac{A_{\mathrm{FF}}}{A_{\mathrm{NF}}},$$

where $I_{tip-in}$ refers to the PL peak intensity with the tip brought into proximity with the sample (TEPL) and $I_{tip-out}$ refers to the PL peak intensity with the tip retracted (far-field PL) from the crystal face. *A*_FF_ and *A*_NF_ indicate the PL measurement areas corresponding to the beam spot created in the focus of an oil-immersion objective lens (NA = 1.25) and the near-field excitation region formed by the Au tip, respectively. The values of $I_{tip-in}$ and $I_{tip-out}$ are derived from curve fitting by a Voigt function. The values of *A_FF_* and *A_NF_* are derived using a general equation for an area of a circle $\pi r^{2}$ by considering a radius $r$_FF_$\simeq\left( \frac{\lambda}{2NA} \right)\times1.5$ = ~380 nm (the empirical factor of 1.5 is used [2]) of the far-field laser spot and $r_{\mathrm{NF}}$ with half of the spatial resolution of the used Au tip, 10 nm (derived from Fig. S4b). Using the EF equation above and the values derived from our experiment (Fig. S4a), we estimate the TEPL enhancement factor for the TEPL without tip-induced sample-modification effects as high as $\sim1.6\times{10}^{3}$.


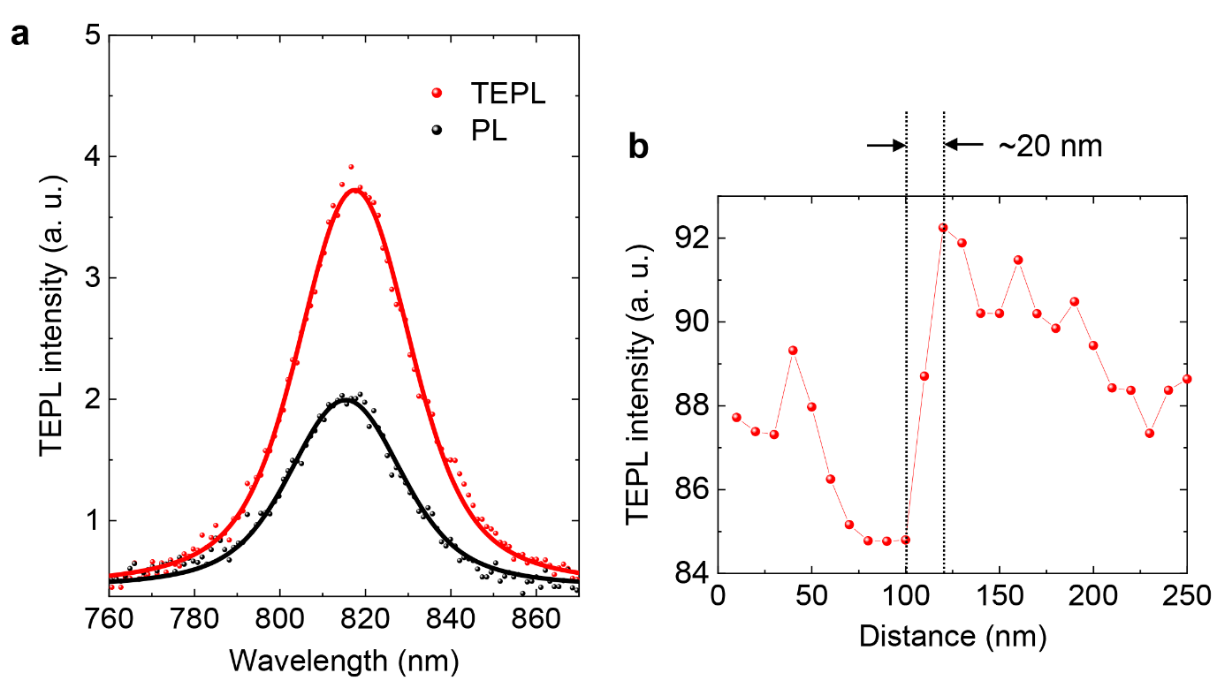


**Fig. S4. a** Comparison of far-field PL and TEPL spectra of IX measured at the region of strong interlayer coupling on the heterobilayer. **b** Line trace of the integrated intensity of the IX TEPL across the L1 as indicated in Fig. 2a in the main text, measured with an Au tip.

5. Reversible tip-induced pressure engineering of interlayer distance in the heterobilayer

First, we place the Au tip at the weak interlayer coupling region to show the tip-induced modifications for three TEPL peaks (IX = 1.52 eV, X_Mo0.5W0.5Se2_ = 1.57 eV, and X_WSe2_ = 1.64 eV), as shown in Fig. S5a. We then slightly press the top layer (WSe_2_) with the Au tip to reduce the interlayer distance *d*_I_ which gives rise to the increased binding energy of IXs by the stronger interlayer coupling at the local region. Fig. S5b shows the resulting TEPL spectrum exhibiting the respective intensity changes of IX, X_Mo0.5W0.5Se2_, and X_WSe2_. As expected, the IX intensity is increased accompanied by the decreased X_Mo0.5W0.5Se2_ and X_WSe2_ intensities, which is attributed to the improved interlayer coupling strength. In this experiment, the tip-induced displacement of the top layer (WSe_2_) is ~0.2 nm, which is precisely regulated by a set-point modulation in a shear-force feedback (see Methods for details). Furthermore, through the simple tip-press and -release process, we can dynamically change the interlayer distance in a reversible fashion without affecting the crystal quality. Fig. S5c shows the TEPL intensity ratios of inter- and intra-layer excitons when the *d*_I_ is changed by the tip-press and -release processes. Both X_Mo0.5W0.5Se2_ and X_WSe2_ show the competing radiative recombination behavior with the IX depending on the interlayer coupling strength.


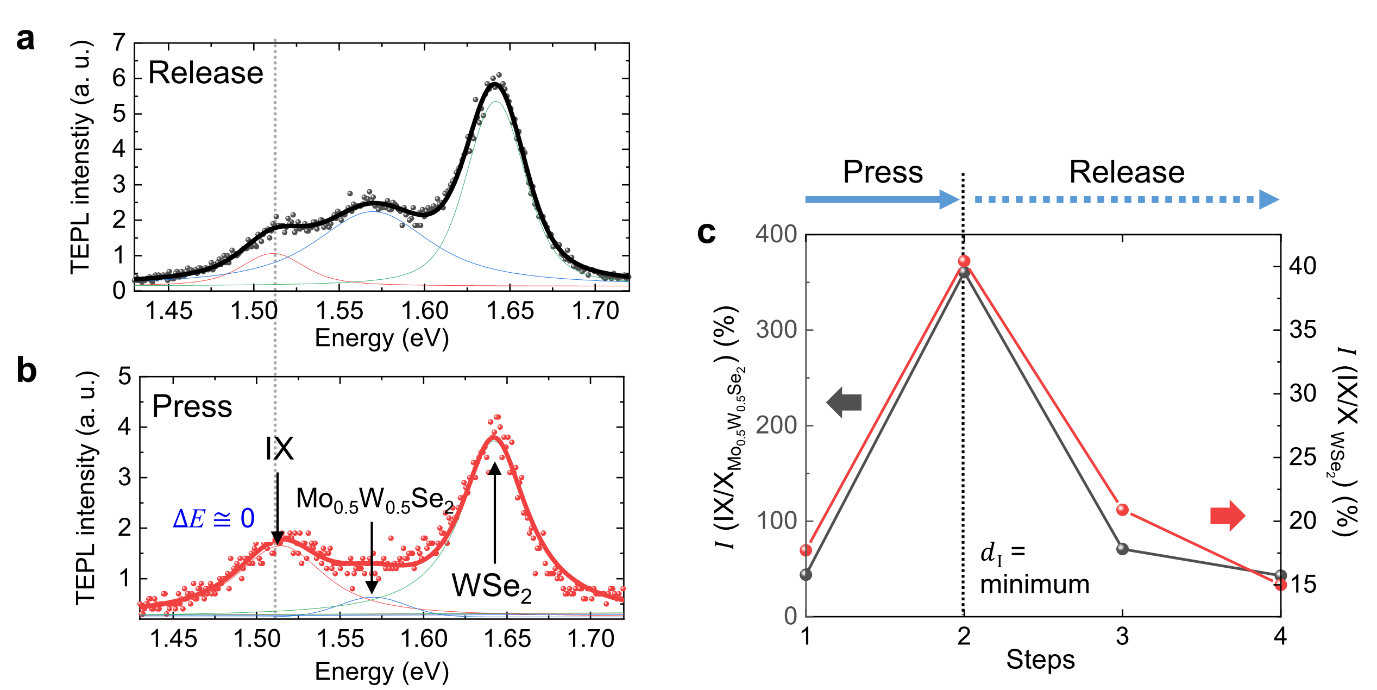


**Fig. S5. a-b.** TEPL spectra obtained at the weak interlayer coupling region before (a) and after (b) pressing the top layer (WSe_2_) with the Au tip to control the interlayer coupling strength. Three TEPL peaks of X_WSe2_, X_Mo0.5W0.5Se2_, and IX are fitted with Lorentzian functions. **c** TEPL intensity ratios of inter- and intra-layer excitons with respect to the interlayer distance changed by the tip-press (steps 1 and 2) and -release (steps 3 and 4) processes.

6. Estimates for the local pressure and force induced by tip-pressure

To estimate the applied pressure during tip-induced bandgap engineering, we calculate the induced local pressure and force using a commercially available numerical simulator (ANSYS) when the tip presses the heterobilayer. Fig. S6 shows the calculated averaged pressure and force near the tip apex region ranging from 0 to 2 GPa and 0 to 40 nN each depending on the pressing depth. The initial interlayer distance is assumed as 0.2 nm for the calculation.


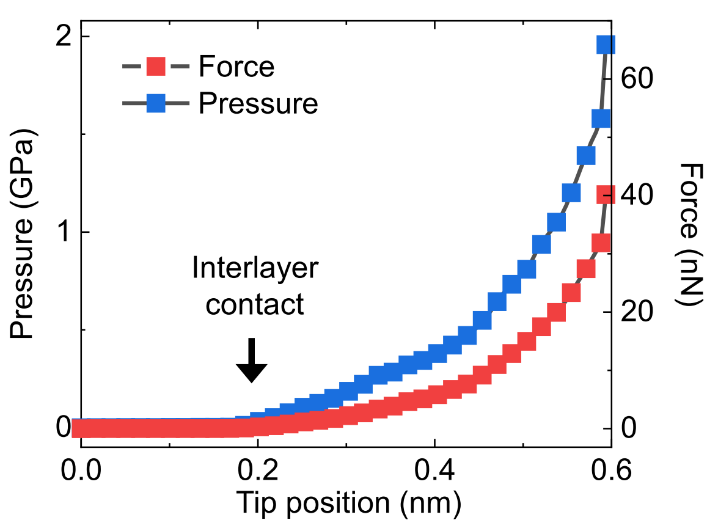


**Fig. S6.** Calculated local pressure and force applied to the heterobilayer when the Au tip presses the heterobilayer.

7. Estimates for the local stress/strain induced by tip-pressure

To quantify the tip-induced local stress and strain at the pressurized heterobilayer, we modeled the Au tip and the WSe_2_/Mo_0.5_W_0.5_Se_2_ heterobilayer using an ANSYS program, as shown in Fig. S7. When the tip-pressure is applied to the top layer, the induced compressive stress ($\sigma$) and strain ($\varepsilon$) reach up to ~12 GPa and ~0.08 % as a maximum value of each, right below the tip apex. However, the tip-induced modification effects are limited to within few nm along the sample surface. By comparing the results of Fig. S3a and b, we can understand that the IX TEPL depends more sensitively on the tip-induced pressure than that corresponding to X_WSe2_ and X_Mo0.5W0.5Se2_.


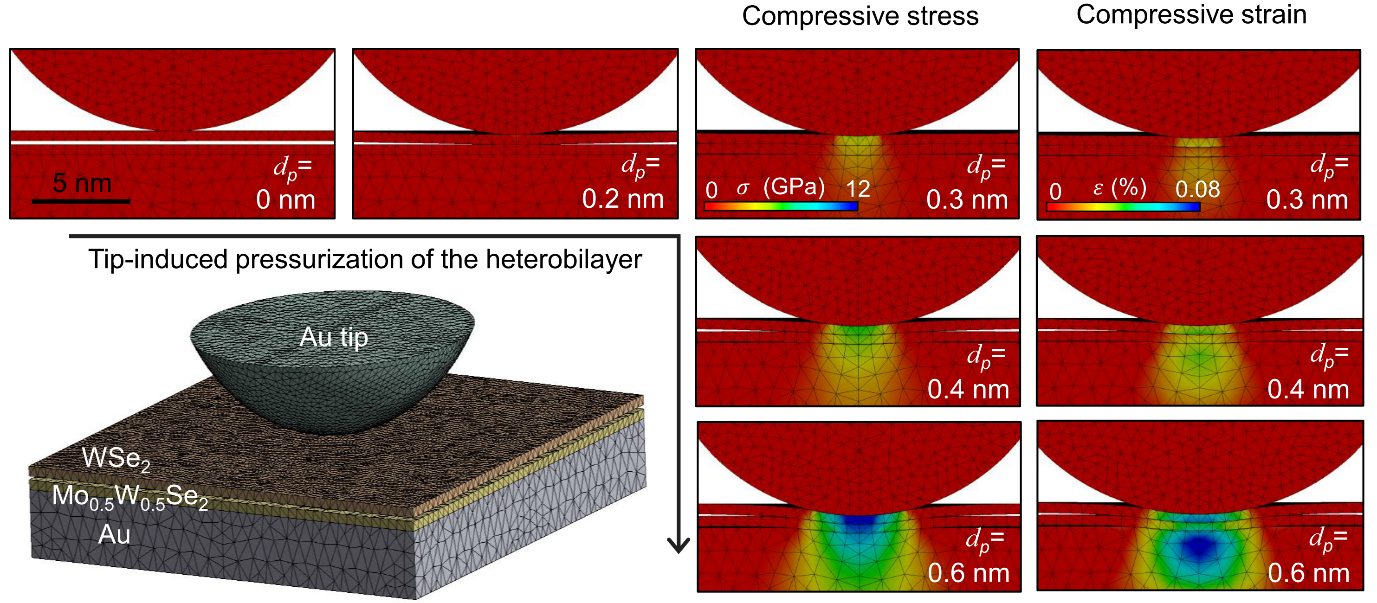


**Fig. S7.** Calculated local compressive stress ($\sigma$) and strain ($\varepsilon$) applied to the WSe_2_/Mo_0.5_W_0.5_Se_2_ heterobilayer on the Au substrate when the Au tip presses for different pressing depths (*d_p_* = 0 to 0.6 nm). Inset figure: Model of the Au tip, Au substrate, and heterobilayer to simulate the tip-induced local deformations of the sample.

8. Calculated strain-induced energy shifts for different W/Mo ratio

For comparison of the simulated results presented in Fig. 3 in main text for the alloy-based heterobilayer with those of pristine TMD bilayers, we calculate bandstructures in a 2$\times$2 supercell geometry and their dependencies on interlayer distance for 3 different compounds: WSe_2_/MoSe_2_, WSe_2_/Mo_0.5_W_0.5_Se_2_, and WSe_2_/WSe_2_. These 3 cases can be considered as different configurations of a general WSe_2_/Mo_1-_*_x_*W*_x_*Se_2_ bilayer with variable W/Mo concentration ratio *x* = 0.0, 0.5, and 1.0. The resulting bandstructures at equilibrium interlayer distance (6.49 Å, 6.45 Å, and 6.42 Å) are shown in Fig. S8a with left, middle, and right panels corresponding to bilayers with *x* = 0.0, 0.5, and 1.0. The calculated total density of states for these 3 structures are shown in (b). Fig. S8c shows the calculated energy shifts at the K-K transition as functions of interlayer distance. While structures with *x* = 0.0 and *x* = 0.5 exhibit blueshift with decreasing interlayer distance, structure with *x* = 1.0 exhibits redshift.


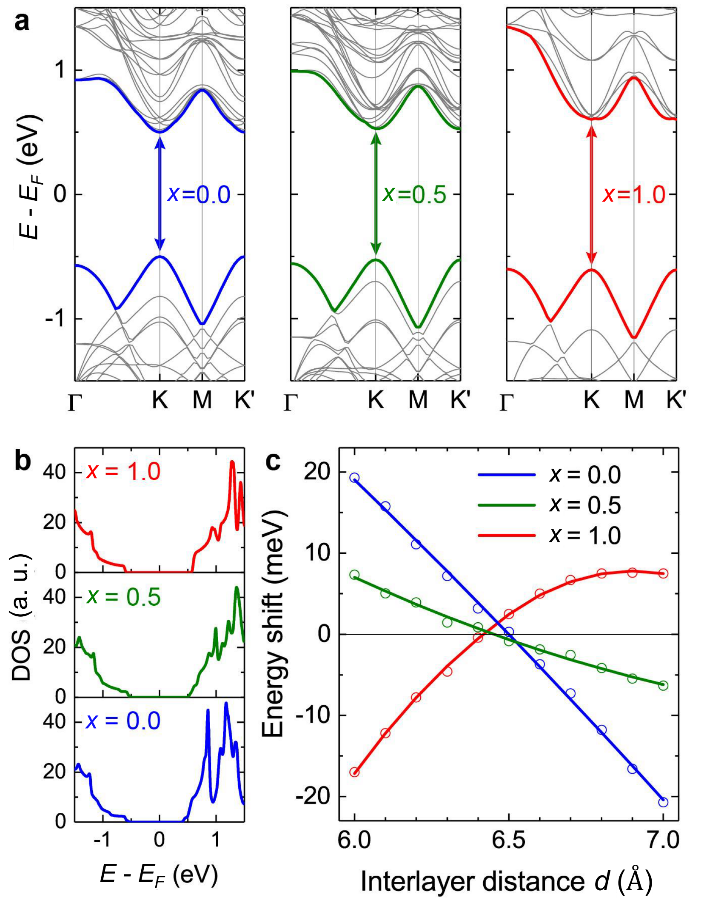


**Fig. S8.** Comparison between DFT calculation results for different values of W/Mo concentration ratio in a general WSe_2_/Mo_1_*_-x_*W*_x_*Se_2_ bilayer. **a** Bandstructures calculated in a 2$\times$2 supercell geometry at equilibrium interlayer distance for *x* = 0.0 (left panel, WSe_2_/MoSe_2_), *x* = 0.5 (middle panel, WSe_2_/Mo_0.5_W_0.5_Se_2_), and *x* = 1.0 (right panel, WSe_2_/WSe_2_). **b** Corresponding total density of states for *x* = 0.0 (lower panel), *x* = 0.5 (middle panel), and *x* = 1.0 (upper panel). **c** Shifts of the K-K transition energy calculated me as functions of interlayer distance for *x* = 0.0 (blue curve), *x* = 0.5 (green curve), and *x* = 1.0 (red curve). Zero shift corresponds to the equilibrium configuration in each case.

9. Determination of the high-power regime for tip-induced hot electron injection

We conduct power dependent measurements of far-field PL of IX and IX- to identify the high-power regime, in which the prominent photocarrier injection from the Au substrate to the heterobilayer appears. As shown in Fig. S9a, we observe almost linear trend of the IX- PL intensity as we increase the excitation power, while the slope of the curve for IX PL decreases with power. This result explains the accelerating process of IX to IX- conversion due to the increasing photocarrier injection. By plotting the intensity ratio of IX- to IX (Fig. S9b), we define two power regimes, i.e., low-power regime and high-power regime, divided at around excitation intensity $\approx$4$\times$10^8^ W m^-2^ where the slope changes rapidly. By comparing two distinct PL spectra from each power regime (Fig. S9c), we can notice that the spectral weight shifts to the lower energy accompanied by the linewidth broadening due to the trion peak emergence at the lower energy side. Our results in Fig. 2 and 3 in the main text were conducted in the low-intensity regime (an order of 10^8^ W m^-2^) to minimize the photocarrier injection effects from the Au tip and substrate. In contrast, the experiments of Fig. 4 in the main text were conducted in the high-intensity regime (an order of 10^9^ W m^-2^) for the effective manipulation of hot electron injection from the Au tip to the heterobilayer.


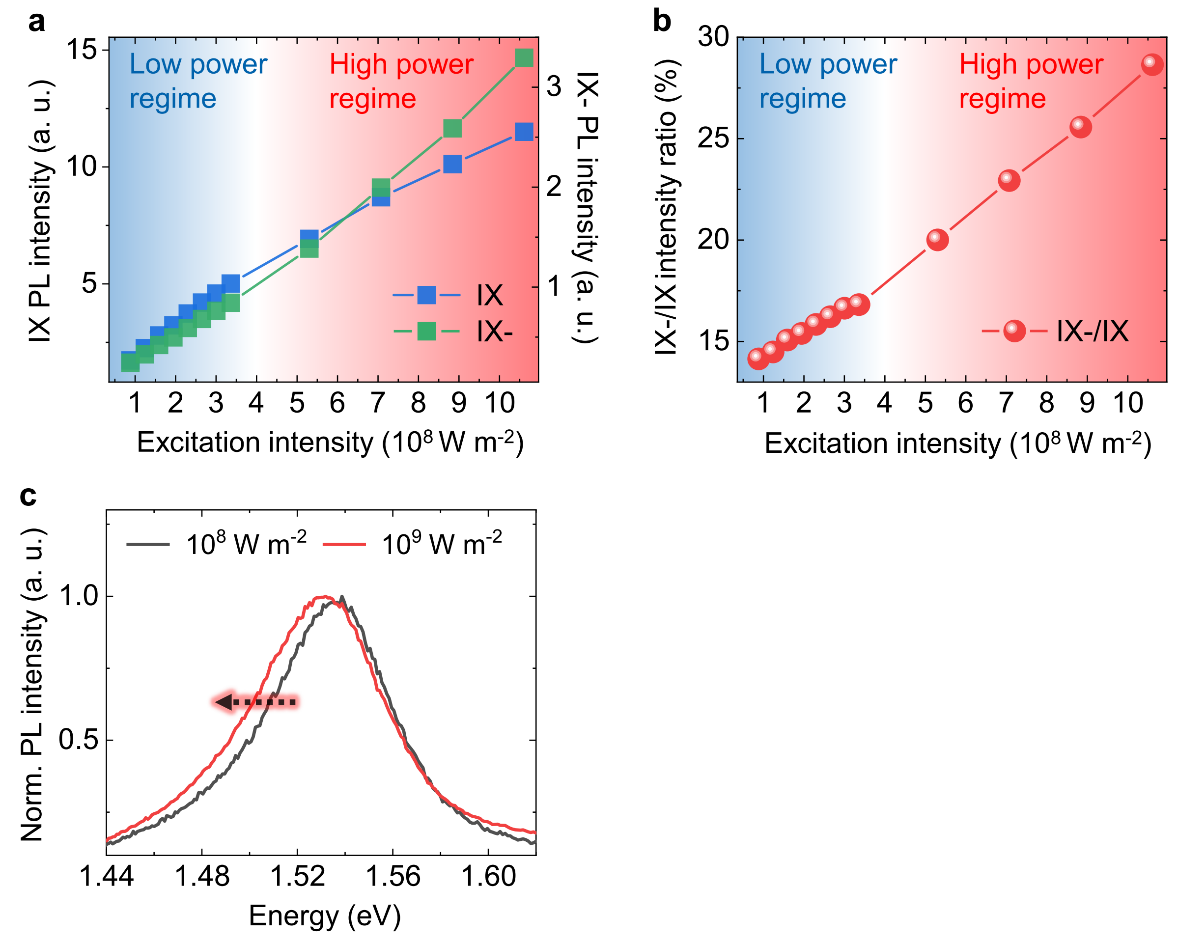


**Fig. S9. a** Evolution of IX (blue) and IX- (green) PL intensity, as a function of excitation power per unit area. All intensities are obtained from Lorentz fits. **b** Evolution of IX- to IX PL intensity ratio showing the increasing conversion rate as the excitation intensity increases. **c** Normalized PL spectra selected from different excitation power regime (10^8^ W m^-2^ for the low-power regime and 10^9^ W m^-2^ for high-power regime) showing significant redshift and linewidth broadening.

10. Reversible pressure-engineering of heterobilayer in the high-power regime favoring IX to IX- conversion

The reversible modification of IX, IX- and X emissions during the tip-induced pressure-engineering under the high-power excitation is demonstrated in Fig. S10. The modified intensity ratio of *I*_IX-_ / *I*_IX_ (red) and *I*_X_ / *I*_IX_ (blue) clearly return to the original state when the tip pressure induced on the heterobilayer is released.


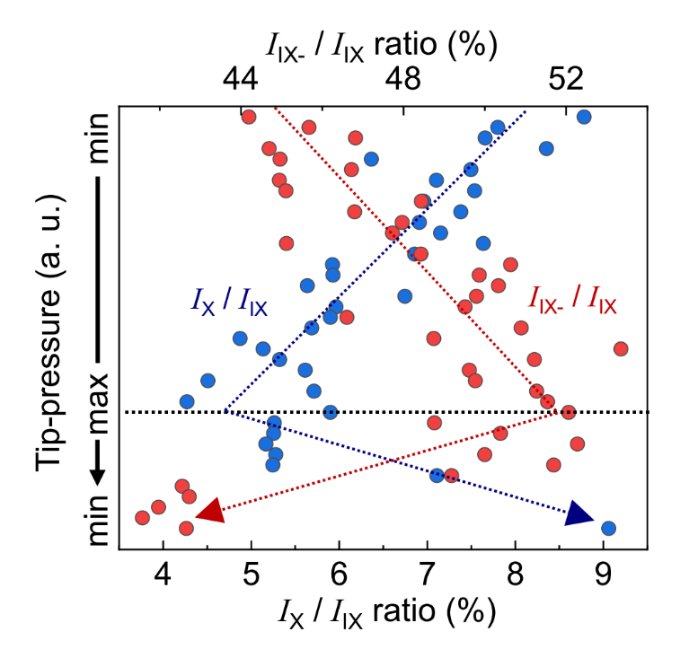


**Fig. S10.** Changes of TEPL intensity ratio of *I*_IX-_ / *I*_IX_ (red) and *I*_X_ / *I*_IX_ (blue) when the Au tip presses and releases the crystal in a reversible manner. Black dashed line indicates the point of maximum pressure.

[1] Stadler, J., Schmid, T. & Zenobi, R. Nanoscale chemical imaging using top-illumination tip-enhanced Raman spectroscopy. *Nano Letters* **10**, 4514-4520 (2010).

[2] Neacsu, C. C., Berweger, S. & Raschke, M. B. Tip-enhanced Raman imaging and nanospectroscopy: sensitivity, symmetry, and selection rules. *NanoBiotechnology* **3**, 172-196 (2007).
